# The Effects of Prenatal Pravastatin Treatment in the Rabbit Fetal Growth Restriction Model

**DOI:** 10.3390/biomedicines11102685

**Published:** 2023-09-30

**Authors:** Katerina Zapletalova, Ignacio Valenzuela, Marnel Greyling, Yannick Regin, Cristian Frigolett, Ladislav Krofta, Jan Deprest, Johannes van der Merwe

**Affiliations:** 1 Department of Development and Regeneration, Cluster Woman and Child, Group Biomedical Sciences, Katholieke Universiteit Leuven, 3000 Leuven, Belgium; katerina.zapletalova@upmd.eu (K.Z.); ignaciovalenzuela7@gmail.com (I.V.);; 2 Institute for the Care of Mother and Child, Third Faculty of Medicine, Charles University, 147 10 Prague, Czech Republic; 3 Department of Public Health and Primary Care, Leuven Statistics Research Centre, Katholieke Universiteit Leuven, 3000 Leuven, Belgium; 4 Department of Obstetrics and Gynecology, Division Woman and Child, University Hospitals Leuven, 3000 Leuven, Belgium

**Keywords:** fetal growth restriction, birthweight, statins, pravastatin, prenatal treatment, rabbit animal model

## Abstract

Fetal growth restriction (FGR) remains without an effective prenatal treatment. Evidence from murine FGR models suggests a beneficial effect of prenatal pravastatin. Since the rabbit hemodichorial placenta more closely resembles the human condition, we investigated the effects of prenatal maternal pravastatin administration in the rabbit FGR model. At a gestational age of 25 days (term 31d), pregnant dams underwent partial uteroplacental vessel ligation (UPVL) in one uterine horn to induce FGR, leaving the other horn as a control. Dams were randomized to either receive 5 mg/kg/d pravastatin dissolved in their drinking water or normal drinking water until delivery. At GA 30d, the rabbits were delivered and were divided into four groups: control without pravastatin (C/NoPrav), FGR without pravastatin (FGR/NoPrav), FGR with pravastatin (FGR/Prav), and controls with pravastatin (C/Prav). The newborn rabbits underwent pulmonary functional assessment and neurobehavioral assessment, and they were harvested for alveolar morphometry or neuropathology. The placentas underwent histology examination and RNA expression. Birth weight was lower in the FGR groups (FGR/Prav, FGR/NoPrav), but there was no difference between FGR/Prav and C/NoPrav. No differences were noted in placental zone proportions, but eNOS in FGR/Prav placentas and VEGFR-2 in FGR/Prav and C/Prav were upregulated. There were no differences in pulmonary function assessment and alveolar morphometry. FGR/Prav kittens had increased neurosensory scores, but there were no differences in neuromotor tests, neuron density, apoptosis, and astrogliosis. In conclusion, in the rabbit FGR model, pravastatin upregulated the expression of VEGFR-2 and eNOS in FGR placentas and was associated with higher neurosensory scores, without measurable effects on birthweight, pulmonary function and morphology, and neuron density.

## 1. Introduction

Fetal growth restriction (FGR) remains to this day one of the main contributors to perinatal mortality and morbidity [1]. Although there is some clinical evidence suggesting the benefit of early postnatal strategies [2,3], no prenatal strategies have accomplished positive clinical results despite dozens of preclinical studies. One of the reasons for the lack of translation from preclinical evidence is the interspecies differences in the therapeutic targets. Placental structure and perinatal organ development from commonly used laboratory animals, i.e., rodents, are far from the human scenario [4].

In the last 15 years, rabbit FGR models have been characterized in great detail. They are attractive because of their close resemblance to humans in terms of their hemodichorial placental structure and major organ development [5]. Specifically, the onset of lung alveolarization prior to birth, and their perinatal brain development [6], increases the translational value of rabbit. In the rabbit FGR model the placental underperfusion induced by uteroplacental vessel ligation (UPVL) leads to a FGR phenotype similar to the human early-onset FGR (EoFGR), i.e., it is associated with a high mortality, multiorgan sequelae, and the accompanying placental histological alterations [7,8,9,10]. The placentas of FGR rabbits show a reduction in the labyrinth zone volume, where the nutrient and gaseous exchange between maternal and fetal blood takes place [11]. Given the known placental alterations seen in human FGR (particularly in early-onset cases), the placenta appears to be the logical target when testing interventions aimed at improving perinatal outcomes. 

Statins have recently gained interest in perinatal research, especially in the context of the amelioration of preeclampsia (PE), a condition that shares certain risk factors and pathophysiology with cardiovascular disease and FGR [12]. Statins upregulate endothelial nitric oxide synthase (eNOS) and induce the expression of antioxidants and anti-inflammatories in a wide range of tissues. They have been shown to ultimately inhibit the release of soluble Fms-like tyrosine kinase (sFlt-1) and soluble endoglin from endothelial cells and placental explants [13,14,15]. 

Previously, pravastatin, a hydrophilic statin, has been shown to increase birth weight and survival in murine preeclampsia models. These effects were mainly brought about by the upregulation of placental vascular endothelial growth factor A (VEFG-A), the restoration of the labyrinth zone fetal capillary volume [16], the induction of the placental growth factor (PlGF) expression [17], and the prevention of placental superoxide production [18]**.** Moreover, in these studies, no adverse effects were observed independent of the model, dosage, or administration route. 

These results have already motivated some clinical trials. In a small observational study, maternal pravastatin treatment did improve the maternal angiogenic profile, lengthen pregnancy duration, and increased the median birthweight in early-onset FGR [19]. Although in a subsequent small multicenter randomized controlled trial, pravastatin treatment did not result in any maternal or fetal benefit when given to women at high risk of preeclampsia [20]. Once again, in both of these studies, no adverse effects were observed. 

The rabbit FGR model seems to be the ideal platform to clarify these ambiguous results. Therefore, we investigated whether prenatal maternally administered pravastatin would impact placental and other organs development. We hypothesize that pravastatin improves birth weight by increasing the placental labyrinth zone proportions. For the first time, we also report the impact thereof on the pulmonary function and alveolar morphometry.

## 2. Materials and Methods

### 2.1. Animal Model

Time-mated rabbit dams (New Zealand rabbit, CEGAV, Saint Marc d’Egrenne, France) were housed in individual cages at 21 °C, 42% humidity, with a 12 h day/night cycle and free access to food and water. Conception day was considered day 0 of pregnancy. At a gestational age (GA) of 25 days (full term 31 days), dams underwent a surgical induction of placental underperfusion by partial UPVL as previously described [21]. Thereafter, dams were randomized using the research randomizer software (version 4.0, retrieved from https://www.randomizer.org/, accessed on 22 May 2022) to receive drinking water with pravastatin (Pravastatine Sodium, Sandoz, Vilvoorde, Belgium) or regular drinking water. Pravastatin was diluted in 250 mL of drinking water to reach a daily dose of 5 mg/kg from GA 25 until delivery, where the dose is based on a previous study on pravastatin effects on antioxidant markers in cholesterol-fed rabbits [22]. Total water intake was monitored throughout, ensuring that each dam received 5 mg/kg/day. 

At GA 30, rabbits were delivered through a caesarian section, and placentas were carefully separated from their implantation sites, trimmed from umbilical cord and membranes, blotted dry, weighed, and divided in two similar portions; one portion was snap-frozen for gene expression analysis, and the other portion was immerse-fixed in 4% paraformaldehyde (PFA) for 72 h. Dams were euthanized using IV phenytoin/pentobarbital (140 mg/kg Euthasol**^®^**, Kela). 

**Figure 1:**
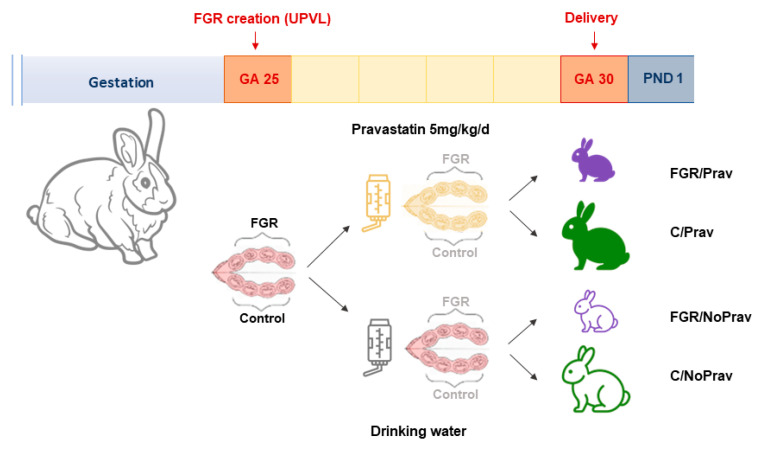
Experimental setup.

Kittens were numbered and kept in a warmed (34 °C) and humidified (55% RH) incubator (TLC-50 Advance, Brinsea**^®^** Products, Weston Super Mare, UK). After four hours, survival was recorded, and they were stimulated to urinate, weighed, and fed a commercial milk substitute (Day One, protein 30%, fat 50%; Fox Valley, Lakemoor, IL, USA) with added probiotics (Bio-Lapis; Probiotics International, Somerset, UK) and immunoglobulins (Col-o-Cat; SanoBest, Hertogenbosch, The Netherlands). On postnatal day 1 (PND 1), litters were allocated to either pulmonary or neurological assessments. Newborn rabbits were divided according to their mother and horn into 4 groups: controls without pravastatin (C/NoPrav), FGR without pravastatin (FGR/NoPrav), FGR with pravastatin (FGR/Prav), and controls with pravastatin (C/Prav). Experimental setup is depicted in Figure 1. 

### 2.2. UPVL Creation

Briefly, rabbits were administered induction anesthesia with IM ketamine (35 mg/kg Nimatek**^®^**, Eurovet Animal Health BV, Bladel, The Netherlands) and xylazine (5 mg/kg XYL-M**^®^** 2%, VMD, Arendonk, Belgium), antibiotic prophylaxis (10 mg/kg enrofloxacin, Baytril**^®^** 2.5% SC, Bayer, Diegem, Belgium), tocolysis (10 mg/kg medroxyprogesterone, Depo-Provera**^®^** SC, Pfizer, Puurs, Belgium), and analgesia (0.03 mg/kg buprenorphine, Vetergesic**^®^** SC, Ceva Animal Health, Brussels, Belgium) prior to surgery. Anesthesia was maintained with a continuous IV infusion of ketamine (8–16 mg/kg/h) and xylazine (2.4–4.8 mg/kg/h) while monitoring vital signs. Following laparotomy, 33–50% of the vessels going to each placenta were ligated in one random horn with Vicryl**^®^** 5–0 (Ethicon**^®^**, Johnson & Johnson, Diegem, Belgium), leaving the contralateral horn as internal control. The abdomen was closed with Vicryl**^®^** 2–0 and Monocryl**^®^** 3–0 (Ethicon**^®^**, Johnson & Johnson) for fascia and skin, respectively. The surgical wound was infiltrated with levobupivacaine (2 mg/kg Chirocaine**^®^**, Abbvie, Wavre, Belgium) and sprayed with aluminum (Kela, Hoogstraten, Belgium). 

### 2.3. Placental Gene Expression

Total RNA was extracted from placental homogenates using the RNeasy mini kit (Qiagen), and cDNA was synthesized using TaqManTM reverse transcription reagents (Thermo Fisher Scientific, Waltham, MA, USA). The expression of catalase (CAT), eNOS, superoxide dismutase (SOD), VEGF-A, and vascular endothelial growth factor receptor 2 (VEFGR-2) was detected using Platinum SYBR Green qPCR Supermix-UDG with ROX (Thermo Fisher Scientific, Waltham, MA, USA). Specimens were run in triplicate and normalized to the housekeeping gene YWHAZ (primers can be found in Table 1).

### 2.4. Placental Histology

After fixation, placentas were paraffin-embedded and cut into 4 um slides. Slides were stained with cytokeratin lectin and scanned with Zeiss AxioScan Z1 imaging platform (AxioScan Slide Scanner, Carl Zeiss MicroImaging GmbH, Munich, Germany). Placental zones (decidua, labyrinth, junction zone) were manually delineated using the QuPath open-source software (version 0.2.0, Belfast, Northern Ireland) [23], and placental zones volumes were calculated from their relative volumes and placental weights as previously described [21]. 

### 2.5. Pulmonary Function Testing (PFT)

On PND 1, pressure-volume and forced oscillation maneuvers were performed using the FlexiVent system (SciReq; FlexiVent, Montreal, QC, Canada) in the first group of kittens. After sedation with ketamine (35 mg/kg) and xylazine (6 mg/kg), a tracheostomy was performed, enabling the insertion of an 18-gauge metal cannula into the trachea. Rabbits were ventilated with a tidal volume of 10 mL/kg and positive end-expiratory pressure of 3 cmH_2_O at a rate of 120 breaths/min. To maximally inflate the lungs and standardize lung volume, two deep inflation maneuvers were performed prior to PFT until reaching a pressure of 30 cm H_2_O. Both pressure-volume (inspiratory capacity, static compliance, and static elastance) and forced oscillation tests (tissue damping, tissue elastance, central airway resistance, respiratory system resistance, dynamic compliance, and dynamic elastance) were performed as previously described [24]. The mean of three separate measurements for each maneuver, with a coefficient of determination >95%, was calculated and used as a single data point for analysis.

### 2.6. Histological Lung Assessment 

After PFT, the lungs were removed via thoracotomy, a 20-gauge catheter was fixed in the trachea, and the left lung was pressure-fixed for 24 h at a constant hydrostatic pressure of 25 cm H_2_O in 4% PFA [24]. After PFA fixation, the left lung was paraffin-embedded and serially cut in 5 µm slides. For alveolar morphometry, 1 slide per lung was stained with hematoxylin and eosin (H&E) and digitally scanned. Mean linear intercept (Lm), alveolar air space (Lma), and interalveolar septal thickness (Lmw) were calculated using a semi-automated, validated Fiji-plugin version 4.0 (ImageJ) (http://fiji.sc/Fiji, accessed on 18 October 2022) [25] that randomly selected 20 fields per lung, according to stereological principles, as previously described [26].

### 2.7. Neurobehavioral Assessment (NBA)

On PND 1, the second group of kittens underwent a validated NBA protocol [6,27]. Short-term motor assessment comprised scoring of gait, posture, locomotion, head and limb activity, and activity duration. Afterward, the cranial nerves, pain response, and righting reflex were tested for sensory evaluation. All assessments were filmed and later scored by an observer blinded to the group assignment (I.V.). A full description of the NBA protocols can be found in the Appendix A (Appendix A.1. Neurobehavioral assessment). 

### 2.8. Brain Harvesting

Immediately after NBA on PND 1, animals were deeply sedated with IM ketamine (35 mg/kg) and xylazine (6 mg/kg), and they were transcardially perfused with 0.9% saline + heparin (100 u/mL; 3 min at 30 mL/min) followed by 4% PFA (5 min at 30 mL/min). Their brains were removed from the skull and further immerse-fixed in 4% PFA for 48 h, and thereafter the brain weight was recorded.

### 2.9. Brain Histology

Following fixation, brains were paraffin-embedded and serially sectioned at 4 µm. Three sets of four serial coronal sections every 100 µm were taken at each of the following two levels, as previously described [6]: level 1 started at the medial septal nucleus and level 2 at the hippocampal formation.

Six slides per brain (three slides per level) were stained with Cresyl Violet (CV; C5042-10G; Sigma- Aldrich, Overijse, Belgium), and two slides per brain (one slide per level) were incubated with each of the following primary antibodies: mouse monoclonal anti-glial fibrillary acidic protein antibody (GFAP) (G6171, Sigma-Aldrich, St Louis, MO, USA), anti-NG2 chondroitin sulfate proteoglycan antibody (MAB5384, Millipore, Billerica, MA, USA), or a terminal deoxynucleotidyl transferase dUTP nick end labeling (TUNEL) method for fluorescent in situ end labeling of double-stranded DNA fragmentation (Apoptag S7110; Millipore). The secondary antibody was Alexa Fluor® 488 goat anti-mouse conjugate (Invitrogen, Sigma-Aldrich, Bornem, Belgium) or Alexa Fluor® 647 goat anti-mouse conjugate. Sections were counterstained with Hoechst 33342 (Sigma-Aldrich, Bornem, Belgium). The following brain areas were assessed: frontal cortex (FC), corpus callosum (CC), caudate nucleus (CN), internal capsule (IC), putamen (P), and hippocampus (HC). 

### 2.10. Statistical Analysis

Sample size calculation was performed using data from previous studies [21] and can be found in Appendix A (Appendix A.2. Sample size calculation). Data were analyzed and graphed using RStudio (Rstudio: Integrated Development for R. Rstudio, PBC, Boston, MA, USA) and GraphPad (GraphPad Prism 9 for MacOS, version 9.4.1, San Diego, CA, USA). The primary outcome was birth weight; all other outcomes were secondary. Data comparison was done using Fisher’s exact for survival, one-way ANOVA for placental RNA, and a linear mixed effects model for all other outcomes. Correction for multiple comparisons was performed using the Tukey method when necessary. Residuals were tested for normality using D’Agostino-Pearson omnibus test. A *p* value of < 0.05 was considered significant. 

## 3. Results

### 3.1. Survival and Biometrics

**Figure 2:**
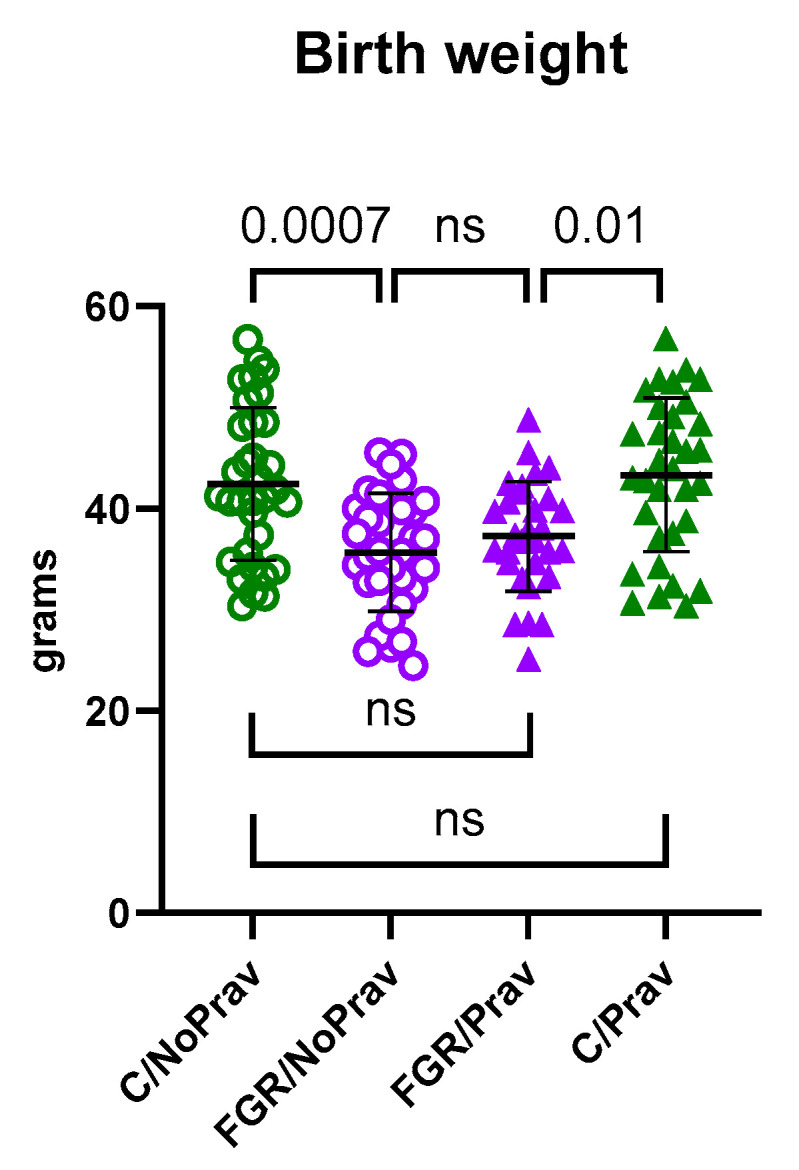
Birth weight comparison of newborn rabbits. C/NoPrav: Controls without pravastatin (33 subjects from 7 litters); FGR/NoPrav: FGR without pravastatin (36 subjects from 7 litters); FGR/Prav: FGR with pravastatin (30 subjects from 7 litters); C/Prav: controls with pravastatin (32 subjects from 7 litters). Data were compared using a linear mixed-model; graph shows mean ± SD; ns (not statistically significant*, p*-value > 0.05).

Birth weight was lower in the FGR/NoPrav as well as the FGR/Prav group when compared to their respective control siblings (Table 2 and Figure 2). Conversely, birth weight from FGR/Prav rabbits was not different from C/NoPrav (*p* = 0.19). Brain-to-body weight ratio was also similar between all groups. Details are shown in Table 2. Survival on PND 1 was similar between all groups.

### 3.2. Placental Histology and Gene Expression

**Figure 3:**
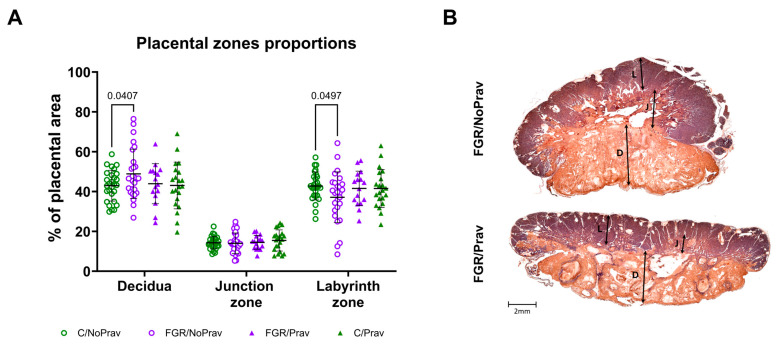
(**A**) Placental zone distribution by histopathological assessment. Data from 87 placentas from 14 litters: C/NoPrav: Controls without pravastatin (32 subjects from 7 litters); FGR/NoPrav: FGR without pravastatin (28 subjects from 7 litters); FGR/Prav: FGR with pravastatin (16 subjects from 7 litters); C/Prav: controls with pravastatin (21 subjects from 7 litters). Data were compared using mixed-effects analysis and are presented as mean ± SD. Only *p*-values < 0.05 are shown. (**B**) Placentas of FGR/NoPrav and FGR/Prav kittens with cytokeratin/lectin double staining, divided by placental zones: decidua (D), junction zone (J), labyrinth (L).

Placentas from all groups did not differ in weight, but the fetal-to-placental weight ratio (FPWR) was significantly decreased in both FGR groups when compared to their respective controls (Table 2). Pravastatin did not alter FPWR in either group. In the histopathological assessment, placentas from FGR/NoPrav had proportionally smaller labyrinth zones than placentas from C/NoPrav (*p* = 0.04; Figure 3). The labyrinth zone proportions were similar between the other groups.

**Figure 4:**
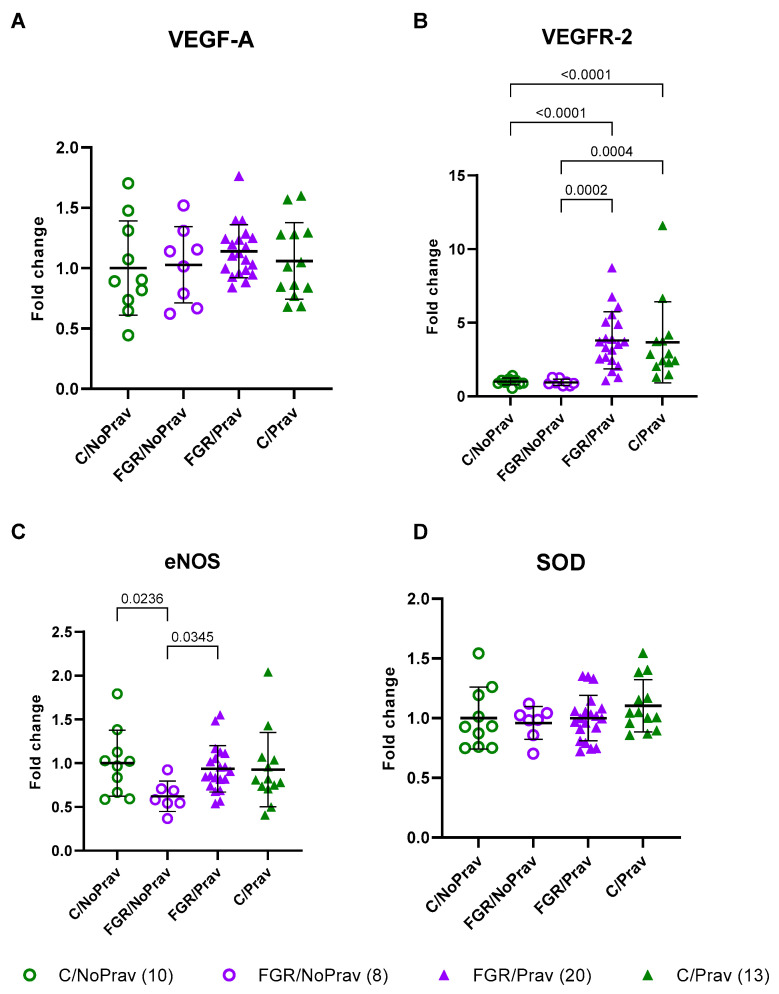
RNA expression of 51 placentas from 7 litters. C/NoPrav: Controls without pravastatin (8 subjects from 4 litters); FGR/NoPrav: FGR without pravastatin (20 subjects from 4 litters); FGR/Prav: FGR with pravastatin (10 subjects from 3 litters); C/Prav: controls with pravastatin (13 subjects from 3 litters). (**A**) VEGF-A: vascular endothelial growth factor; (**B**) VEGFR-2: vascular endothelial growth factor receptor 2; (**C**) eNOS: endothelial nitric oxide synthase; (**D**) SOD: superoxide dismutase;. Data analyzed using one-way ANOVA, shown as mean ± SD. Only *p*-values < 0.05 are shown.

The expression of CAT, SOD, and VEGF-A in FGR/NP placentas was similar to that of C/NoPrav placentas. Pravastatin increased the expression of VEGFR-2 in both FGR and controls. Expression of eNOS was decreased in FGR/NoPrav placentas when compared to C/NoPrav (*p* = 0.02). Pravastatin significantly increased eNOS expression in FGR placentas (*p* = 0.03) when compared to FGR/NoPrav (Figure 4). 

### 3.3. Pulmonary Function and Structure

Lungs from untreated FGR rabbits showed increased tissue elastance (*p* = 0.001), reduced hysteresis (*p* = 0.01), dynamic compliance (*p* = 0.02), and static compliance (*p* = 0.03) when compared to untreated controls. Increased tissue damping (*p* = 0.06) and respiratory system resistance (*p* = 0.1) were not different between these two groups. Pravastatin had no effect on pulmonary function in any of the treated groups (Table 3). 

**Figure 5:**
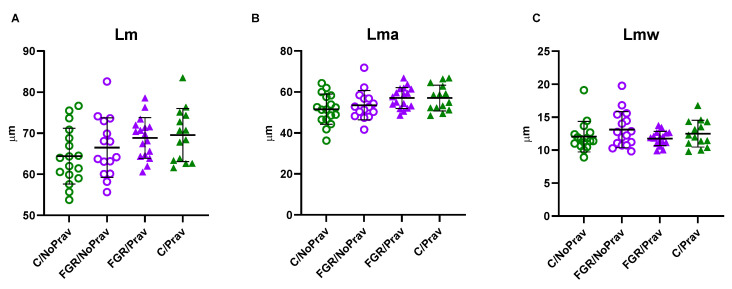
Alveolar morphometry: (**A**) Alveolar size (Lm), (**B**) Alveolar airspace, (**C**) Alveolar wall thickness (Lmw) from 63 newborn rabbits from 8 litters. Data comparison was done using a mixed-effects model, with correction for multiple comparison using Tukey test. Data are presented as mean ± SD. C/NoPrav: Controls without pravastatin (17 subjects from 4 litters); FGR/NoPrav: FGR without pravastatin (16 subjects from 4 litters); FGR/Prav: FGR with pravastatin (17 subjects from 3 litters); C/Prav: controls with pravastatin (14 subjects from 3 litters).

In the histological assessment, neither FGR nor pravastatin had a significant effect on alveolar size (Lm), alveolar airspace (Lma), or alveolar wall thickness (Lmw; Figure 5).

### 3.4. Neurobehavioral and Neuropathological Findings

**Figure 6:**
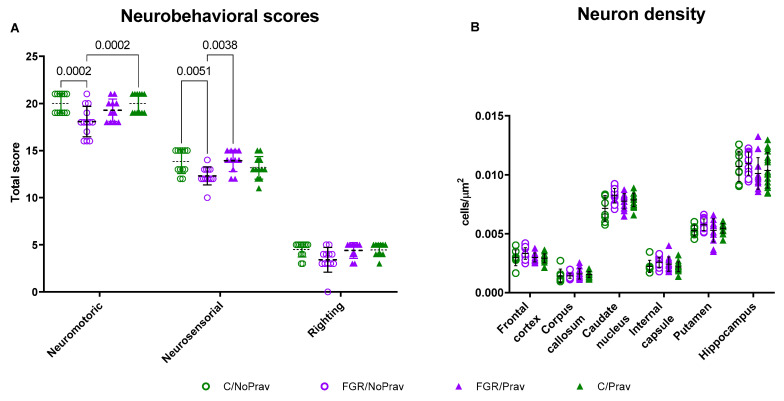
Neurodevelopmental assessment at PND 1 in postnatal day 1 rabbits. (**A**) Neurobehavioral tests grouped scores. (**B**) Cell density assessed in cresyl violet stained slides. Data were analyzed using a linear mixed-effects model and are displayed mean ± SD.

Neuromotor and neurosensory scores were lower in the FGR/NoPrav group when compared to C/NoPrav (Figure 6A). Neuromotor scores from FGR/Prav were not different from FGR/NoPrav (*p* = 0.06), but there was a higher total score in the FGR/Prav neurosensory tests (*p* = 0.0004). Righting reflex was comparable in all the four groups. 

Neuron density was comparable between all groups in FC, CC, CN, IC, putamen, and hippocampus (Figure 6B, Table A1). Apoptosis and astrogliosis were also comparable between groups in these regions (Table A1). 

## 4. Discussion

In the rabbit FGR model, FGR rabbits’ birthweight remains lower despite the maternal prenatal pravastatin administration from GA 25d, the date of UPVL. Also, no other measurable effect in pulmonary or neuropathology readouts were observed. However, pravastatin was associated with higher neurosensory scores at PND 1 and an upregulation of eNOS and VEFGR-2 in pravastatin-exposed placentas. 

The vascular production of nitric oxide (NO), a vasoprotective and vasodilator molecule, is mainly induced by eNOS [28]. During pregnancy, eNOS is crucial for maternal cardiovascular adaptations and the regulation of uterine and fetoplacental circulation [29]. Moreover, placental metabolism is dependent on the proper functioning of this enzyme [30], and placentas from pregnancies with FGR have been found to have a reduced expression of eNOS [31,32]. Furthermore, the experimental inhibition of eNOS by either genetic knockout or pharmacologic inhibition leads to FGR and PE-like phenotype in several animal models [33,34,35]. Interestingly, surgically induced placental underperfusion in this UPVL rabbit model decreased eNOS RNA in the placenta. Pravastatin normalized the expression of eNOS to the levels measured in healthy non-pravastatin-exposed controls. Statins have been shown to increase the expression and function of eNOS through several different mechanisms and pathways, including the activation of the AMP-activated protein kinase and Akt signaling pathways, and the inhibition of the Rho/ROCK pathway [36,37]. The specific pathway that is activated or inhibited in this model, and the result in eNOS protein concentration and activation, are interesting subjects for a future expansion of the model. 

VEGF is a glycoprotein with potent angiogenic properties; it exerts its effects by binding to two receptors: VEGFR-1 (fms-like tyrosine kinase-1 or flt-1) and VEGFR-2 (fetal liver kinase-1 or flk-1), both of which have two isoforms: soluble or membranous. The soluble form of Flt-1 (sFlt-1) is enhanced in EoFGR [38,39] to such a degree that it is used in detection and management of EoFGR [40,41]. This isoform binds to VEGF with 10 times the affinity of VEGFR-2, leading to inhibition of its biological activity and subsequent endothelial disfunction. In human placentas, VEGFR-2 is localized almost exclusively on the endothelial cells of the blood vessels of placental villi [42], and its expression appears to remain unchanged in pregnancies complicated by FGR [42]. In accordance with evidence, we did not find differences between our untreated FGR and controls in VEGFR-2 expression. On the other hand, a 4–5-fold increase in VEGFR-2 expression was observed after pravastatin administration in both FGR and controls. Interestingly, no significant differences were found in the expression of its ligand, VEGF-A. The protein levels of these two molecules, along with VEGFR-1 and its soluble isoform, will potentially inform us on the plausible mechanism behind these findings. In addition, as with eNOS expression, the pathways behind this effect should be further elucidated in this model. 

Pravastatin, given its hydrophilic properties, exerts its main effects in maternal and placental tissues [43,44]. Thus, the improvement of fetal or neonatal outcomes would likely be the result of an improvement in placental function. In our study, this was not the case, as our primary outcome, birth weight, was not improved by prenatal pravastatin. Additionally, no differences were observed in the secondary readouts such as pulmonary function, alveolar morphometry, or neuron density in the brain. It is noteworthy that the birth weight observed in the untreated FGR group was higher than in previous reports of this model and that model heterogeneity is an inherent limitation in most preclinical research. On the other hand, pravastatin was associated with a small but significant increase in neurosensory scores. Although this study was not powered to detect small effects on secondary outcomes, these findings warrant further investigation.

We acknowledge that our study has limitations. Firstly, the number of animals in each group was determined for the primary outcome. Other significant differences in secondary outcomes might have been missed due to the lower effect size or number of subjects. Secondly, the terminal nature of our experiments did not allow for a long-term or longitudinal examination. Lastly, the birth weight in the untreated FGR was higher than previously reported, which may have contributed to the lack of significant findings. However, the birth weight was still significantly lower than in the controls, and the untreated FGR was associated with worse placental and pulmonary outcomes. 

## 5. Conclusions

According to the results of this study, in the rabbit UPVL model maternal prenatal pravastatin administered after FGR induction does not improve birth weight. However, an upregulation of angiogenic gene expression in the placenta was noted, which could indicate that in a less severe FGR model, it would have had a wider range of effects. 

## Figures and Tables

**Table 1:** Primers used in this study.

Gene	Position	Primer Sequence
*CAT*	I3	ACCCCCATTGCAGTTCGATT
	I4	CCGGGTCCTTTAGGTGTGTC
*eNOS*	C1	ACAGTTACCAGCTCGCCAAA
	C2	GCTCATTCTCCAGGTGCTTC
*SOD*	H3	GACGCATAACAGGACTGACCG
	H4	AACACATCAGCGACACCATTG
*VEGF-A*	F1	CTTGCTGCTCTACCTCCACC
	F2	CTTTGGTCTGCATTCACATTTG
*VEGFR-2*	G1	CCCCTGATTACACTACGCCC
	G2	TGTAGTCTTTGCCACCCTGC
*YWHAZ*	H8	GGTCTGGCCCTTAACTTCTCTGTGTTCTA
	H9	GCGTGCTGTCTTTGTATGATTCTTCACTT

**Table 2:** Survival and biometrics. Newborn rabbits from 14 dams: 7 in the Prav group and 7 in the NoPrav group. N shows the total number of newborn rabbits in each group.

Group	C/NoPrav	FGR/NoPrav	FGR/Prav	C/Prav
Survival at birth	39/40 (98%)	38/43 (88%)	34/41 (83%)	35/39 (90%)
Survival at PND 1	33/39 (84%)	36/43 (83%)	30/41 (73%)	32/39 (82%)
Birth weight (g)	42.73 ± 1.48	35.99 ± 3.16 ^ab^	37.50 ± 6.67 ^b^	43.09 ± 3.57
Placental weight (g)	6.04 ± 0.33	5.63 ± 0.54	5.44 ± 1.3	5.60 ± 0.81
BBWR	0.045 ± 0.003	0.049 ± 0.002	0.046 ± 0.003	0.048 ± 0.004
FPWR	7.23 ± 0.30	6.52 ± 0.52 ^c^	7.06 ± 1.2 ^b^	7.75 ± 0.73

Data were analyzed using linear mixed-model and expressed as *n* (%) or mean ± SD. BBWR: brain-to-body weight ratio; C/NoPrav: Controls without pravastatin; FGR/NoPrav: FGR without pravastatin; FGR/Prav: FGR with pravastatin; C/Prav: controls with pravastatin; FGR: fetal growth restriction; FPWR: fetal-to-placental weight ratio; PND 1: postnatal day 1. ^a^ *p* < 0.001 when compared to C/NoPrav. ^b^ *p* < 0.05 when compared to C/Prav. ^c^ *p* < 0.01 when compared to C/NoPrav.

**Table 3:** Pulmonary function tests.

Parameter	Control/NoPrav (*n* = 17)	FGR/NoPrav (*n* = 16)	FGR/Prav (*n* = 18)	Control/Prav (*n* = 15)
Inspiratory capacity, mL/kg	32.07 ± 2.68	27.96 ± 4.72	24.70 ± 7.45 ^b^	31.7 ± 6.52
Static compliance mL/(cm H_2_O·kg)	2.58 ± 0.23	2.12 ± 0.38	1.87 ± 0.61	2.27 ± 0.55
Hysteresis (A), mL·cm H_2_O	1.44 ± 0.13	1.11 ± 0.23 ^c^	1.02 ± 0.54 ^b^	1.37 ± 0.30
Tissue elastance (H), cm H_2_O/mL	8.35 ± 1.19	12.39 ± 2.18 ^d^	10.58 ± 3.56 ^a^	7.62 ± 2.91
Tissue damping (G), cm H_2_O/mL	2.31 ± 0.26	2.95 ± 0.51	2.89 ± 0.86 ^a^	2.17 ± 0.65
Respiratory system resistance, cmH_2_O·s/mL	0.297 ± 0.034	0.380 ± 0.071	0.357 ± 0.121	0.302 ± 0.083
Central airway resistance, cmH_2_O·s/mL	0.099 ± 0.028	0.085 ± 0.055	0.055 ± 0.13	0.035 ± 0.068
Dynamic compliance, mL/cmH_2_O·kg	2.15 ± 0.23	1.70 ± 0.38 ^a^	1.55 ± 0.91	1.94 ± 0.55
Inspiratory capacity, mL/kg	32.07 ± 2.68	27.96 ± 4.72	24.70 ± 7.45 ^b^	31.7 ± 6.52
Static compliance mL/(cmH_2_0·kg)	2.58 ± 0.23	2.12 ± 0.38	1.87 ± 0.61	2.27 ± 0.55
Hysteresis (A), mL·cmH_2_O	1.44 ± 0.13	1.11 ± 0.23 ^c^	1.02 ± 0.54 ^b^	1.37 ± 0.30
Tissue elastance (H), cmH_2_O/mL	8.35 ± 1.19	12.39 ± 2.18 ^d^	10.58 ± 3.56 ^a^	7.62 ± 2.91
Tissue damping (G), cmH_2_O/mL	2.31 ± 0.26	2.95 ± 0.51	2.89 ± 0.86 ^a^	2.17 ± 0.65
Respiratory system resistance, cmH_2_O·s/mL	0.297 ± 0.034	0.380 ± 0.071	0.357 ± 0.121	0.302 ± 0.083
Central airway resistance, cmH_2_O·s/mL	0.099 ± 0.028	0.085 ± 0.055	0.055 ± 0.13	0.035 ± 0.068
Dynamic compliance, mL/cmH_2_O·kg	2.15 ± 0.23	1.70 ± 0.38 ^a^	1.55 ± 0.91	1.94 ± 0.55

Inspiratory capacity, static compliance, and dynamic compliance are corrected by body weight. C/NoPrav: Controls without pravastatin; FGR/NoPrav: FGR without pravastatin; FGR/Prav: FGR with pravastatin; C/Prav: controls with pravastatin; FGR: fetal growth restriction. ^a^ *p* < 0.05 versus C/Prav; ^b^ *p* < 0.01 versus C/Prav; ^c^ *p* < 0.05 versus C/NoPrav; ^d^ *p* < 0.01 versus C/NoPrav.

## Data Availability

The datasets analyzed during the current study are available from the corresponding author upon reasonable request.

## Appendix A

### Appendix A.1. Neurobehavioral Assessment

The neurobehavioral PND1 evaluation is based on a modification of neurobehavioral scoring protocol previously described [27]. For each animal, testing is videotaped and scored by a blinded observer. The kittens are evaluated in a designated space close to their pen with auditory and olfactory contamination kept to a minimum. Before handling, they remain undisturbed in this assessment area for a 3–5 min adaptation period.

- Cranial nerves are assessed by testing smell (olfaction is tested by recording time to aversive response to a cotton swab soaked with pure ethanol), sucking, swallowing (by introduction of formula into the kittens’ mouth with a plastic syringe), and head turn to feeding. The responses are graded on a scale of 0 to 3, 0 being the worst response and 3 the best response.
- Motor examination includes tone, motor activity, locomotion on a flat surface, righting reflex, and gait. The righting reflex is assessed when the kittens are placed on their backs, and the number of times turned prone (within 2 s) from supine position in 5 tries is registered. Gait is examined based on a modification by Georgiadis et al. [46]. Locomotion is assessed as described by Kannan et al. [47].
- Sensory examination is limited to touch on the face (touching the face with cotton swab on both sides) and extremities as well as pain on limbs (mild pin prick).

### Appendix A.2. Sample Size Calculation

**Input**:	Tail (s)	=	Two
	Effect size d	=	0.7243715
	α err prob	=	0.05
	Power (1-β err prob)	=	0.8
	Allocation ratio N2/N1	=	1
**Output**:	Noncentrality parameter δ	=	2.8518534
	Critical t	=	2.0002978
	Df	=	60
	Sample size group 1	=	31
	Sample size group 2	=	31
	Total sample size	=	62
	Actual power	=	0.80121

**Table A1:** Neuropathological assessment in postnatal day 1 brains.

Parameter	Control/NoPrav (*n* = 14)	FGR/NoPrav (*n* = 17)	FGR/Prav (*n* = 13)	Control/Prav (*n* = 17)
Neuron density (cells/μm^2^)				
Frontal cortex	0.0029 ± 0.0001	0.0032 ± 0.0002	0.0030 ± 0.0.0003	0.0029 ± 0.0002
Corpus calosum	0.0014 ± 0.0002	0.0014 ± 0.0002	0.0015 ± 0.0002	0.0015 ± 0.0002
Caudate nucleus	0.0070 ± 0.0002	0.0081 ± 0.0003	0.0077 ± 0.0004	0.0060 ± 0.0002
Internal capsule	0.0021 ± 0.0003	0.0024 ± 0.0002	0.0024 ± 0.0004	0.0021 ± 0.0002
Putamen	0.0051 ± 0.001	0.0056 ± 0.001	0.0080 ± 0.002	0.0071 ± 0.002
Hippocampus	0.011 ± 0.0004	0.011 ± 0.0005	0.010 ± 0.0006	0.010 ± 0.0006
GFAP (+) cells (%)				
Frontal cortex	0.18 ± 0.17	0.37 ± 0.17	0.21 ± 0.25	0.22 ± 0.25
Corpus calosum	26.66 ± 9.7	28.88 ± 3.85	30.79 ± 13.99	28.75 ± 5.78
Caudate nucleus	0.20 ± 0.06	0.09 ± 0.08	0.06 ± 0.12	0.09 ± 0.10
Internal capsule	0.24 ± 021	0.22 ± 0.22	0.41 ± 0.32	0.50 ± 0.31
Putamen	0.051 ± 0.09	0.31 ± 0.11	0.086 ± 0.16	0.12 ± 0.12
Hippocampus	1.04 ± 1.60	1.78 ± 0.79	2.10 ± 1.18	1.92 ± 2.31
TUNEL (+) cells (%)				
Frontal cortex	0.12 ± 0.06	0.14 ± 0.04	0.033 ± 0.05	0.078 ± 0.06
Corpus calosum	1.06 ± 0.36	1.21 ± 0.24	0.93 ± 0.33	0.83 ± 0.46
Caudate nucleus	0.32 ± 0.13	0.58 ± 0.15	0.25 ± 0.21	0.33 ± 0.16
Internal capsule	0.26 ± 0.010	0.33 ± 0.13	0.35 ± 0.17	0.26 ± 0.12
Putamen	0.73 ± 0.35	0.93 ± 0.30	0.35 ± 0.4	0.42 ± 0.44
Hippocampus	0.057 ± 0.07	0.289 ± 0.09	0.12 ± 0.12	0.074 ± 0.09
